# Retraction Note: The value of subcutaneous tissue closure and drain in obese women undergo elective caesarean section: a randomized controlled trial

**DOI:** 10.1186/s12884-026-09230-x

**Published:** 2026-05-08

**Authors:** Mohamed A. Shalaby, Hanan H. M. Metwally, Ahmed M. Maged, Yomna A. Bayoumi, Noha Salah

**Affiliations:** 1 Department of Obstetrics and Gynecology, Kasr Al-Ainy Hospital, Cairo University, Cairo, Egypt; 2 Department of Obstetrics and Gynecology, Temi Elamdid Hospital, Mansoura, Dakahlia Egypt

**Retraction Note: BMC Pregnancy and Childbirth 25, 534 (2025)**

**https://doi.org/10.1186/s12884-025-07579-z**

The Editor has retracted this article. Concerns were raised about the presence of duplicate combinations in the data and abnormal distribution of BMI. Post-publication review confirmed the issues. The Editor has therefore lost confidence in the findings of this article. Mohammed Shalaby has stated on behalf of all authors that they do not agree to this retraction.

